# Discussion

**Published:** 1918-08

**Authors:** 


**Discussion :**

Dr. Tom A. Williams said that there are two chief clinical ways in which psychological disturbances have shown themselves during the present war. The first is motor impotence. In most of these cases there has been a wound; often, however, only slight; but the patient does not recover utility when the wound gets well. The second is a general nervousness without lesion, usually stated by the patient to be the result of what he calls "shell-shock".

In both of these conditions, the first duty of the neurologist is to make sure that physical disturbances are not responsible for any part of the condition. In peripheral disorders, when he has discovered the integrity of the muscles by their electric reactions and has shown by warming the limb that the circulation is intact; and when he has established the freedom of the articulations from adhesions, he is then well in a position to affirm that the patient's disability is purely psychic; and that it is completely removable by the methods he knows how to apply.

n the so-called " shell-shock " cases, the neurologist will make sute that there is no alteration of the cerebro-spinal fluid, that the vasomotor system has not been put out of gear, that the reactional movements provoked by labyrinthine stimulus are normal, and, after doing so, he will be prepared to enter into the psychogenetic factors of the patient's illness and to deal with the patient by methods not dissimilar in principle to those applied to the cases of peripheral incapacity.

The clinician must remember the great difference of resistance in patients. Their threshold to stimuli varies as regards :

1. The bearing of pain, every surgeon knowing how some patients will withstand the most painful dressings, while others writhe in agony. This is not only dependent upon psychological fortitude, but upon the physical state of the organism. After loss of blood, starvation, during suppuration and shock, resistance is greatly lowered. Some persons again seem to be constitutionally less susceptible to pain.

2. The threshold to emotive disturbances is most variable.

3. The most variable of all factors, however, is that of suggestibility. Most persons, however, are extremely suggestible.

It is upon the ductility of humanity that the neurologist depends for his success in returning psychoneurotics to service. For the same facility with which the man becomes a psychoneurotic favors his return to normal. However, the measures used must be properly designed and skilfully carried out, and, if so, one or two sittings suffice for cure, which is really easy.

Very unequal have been the results obtained by different psychiatrists and in different countries. Zeal and wholeheartedness are indispensable qualifications, but they are inadequate unless based upon correct diagnoses and utilized in conformity with the actual mechanism of the patient's disorders. Again the psychotherapeutist must not only himself be skilful, but must provide an atmosphere in the hospital which bears upon the reeducation of the men which he seeks to effect.

This is most easily provided in the Army Zone, and under strict military discipline; for in this situation the dangers of contrary influences are minimized. In the Army Zone, a doctor has all the prestige of an officer of the army, disobedience to whose orders is a serious infraction of military discipline and bears all the consequences of such a procedure.

Thus there are various methods of obtaining the effect to be aimed at, namely, the impression of the patient by the certainty that he can be cured. Of course, in spite of all methods, this suggestion often fails, and then the work must be done entirely by the doctor himself. This consists in a proper analysis of the mechanism of the morbid affective state.

The whole treatment is likely to be taken exception to by the friends of patients, by self-seeking demagogues, or by misled philanthropists. Thus the methods that are required must have the support of public opinion. This, of course, depends upon medical opinion, which is dependent upon neurological doctrines regarding the mechanism of the psychoneuroses.

Lieut.-Colonel Gordon Holmes, R. A. M. C., dealt briefly with the treatment and management of the psyschoneuroses as they appear in combatant Armies, rather than with their clinical symptoms and nature, as success is most certainly assured when adequate clinical methods can be rationally employed under suitable conditions.

The importance of this subject lies not only in the serious wastage to the combatant forces that may accrue if successful steps are not taken to check the development of neurotic states and to relieve as quickly as possible the functional and nervous symptoms which appear, but it is also a matter of great social importance too, because, if the cases are not treated successfully, they are liable, when returned to civil life, to degenerate into social parasites and become a moral incubus as well as a financial burden on their country. The latest statement made in the British Parliament revealed the fact told that 6 o/o of those pensioned, or over 20,000 men, have been invalided from British armies with " shell shock ", and common experience under the Compensation Act makes it probable that a large proportion of these will remain civil pensioners and unproductive units to the State. The great majority of these men were certainly evacuated from the army before it was recognized that early and vigorous treatment under rigid military discipline is essential, and that the proportion of recoveries is very much smaller when active therapeutics is delayed.

It was not till the winter of 1916 that our present system was brought into operation by the present Director General. The French had already recognized that the first essential step was to establish neurological centers within the army areas, as near the front as was compatible with safety and suitable conditions, where the men could come under treatment before their symptoms became fixed and organized, and could remain under the control of the armies to which they belonged. Similar centers are established in each British army; into these all the cases evacuated from the medical units are transferred as early as possible. The accommodation of each center must vary with the number of troops within the army area and the severity and nature of the operations in which the army may be engaged, but should be sufficiently large to assure that all who may recover within a reasonable time can be returned directly from them to their units. This is always desirable as there is a considerable danger of the recurrence or remission of symptoms when such patients are transferred through a series of hospitals, convalescent camps, and base dépôts, and come under the care of officers who may not be fully acquainted with their previous conditions.

As a certain proportion of the patients must be eventually evacuated there should also be centers or specially organized divisions in the base hospitals on the lines of communication to receive them. These cases comprise men with severe symptoms, chiefly those with an unstable emotional basis, and patients who have already been treated for some manifestation of the psychoneuroses and have broken down again after a relatively short period under fire. Many of these eventually recover and become again fit for service, and most of the others can usefully be employed in base work. The percentage that under this system must be evacuated to England, or be permanently lost to the Army, is consequently very small.

Success in the treatment of these cases depends chiefly on the organization of the army neurological centers. No elaborate hospital accommodation is needed — some of the most satisfactory work has been carried out under canvas. As strict isolation is necessary in a small proportion of cases, especially in those with tremors and irregular tic-like movements, a certain number of small isolation cubicles should be provided, and there should be, of course, quiet examination and treatment rooms where each man can be examined and dealt with alone. Provided the centre is properly managed and the medical staff is efficient and creates the proper atmosphere, these functional cases recover much more quickly and certainly when concentrated in such centers than when they are scattered among the sick and wounded.

The staff of each Center should include at least one competent neurologist, or at least a medical officer with sufficient training and experience in nervous diseases to diagnose and recognize with certainty the various neuropathic conditions that he may meet. As

Major Kennedy said, everyone recognizes that the primary essential in treating functional nervous disorders is self-confidence on the part of the medical officer, for unless he is a mere charlatan a doctor cannot inspire the patients with confidence if he is uncertain whether they are suffering from functional or organic conditions. It must not be forgotten that men suffering with injuries of the head and spinal cord as well as others suffering with tabes, general paralysis, disseminated sclerosis, and other organic conditions, will frequently be admitted to your neurological centers.

Colonel Salmon mentioned in his address that the American Expeditionary Force proposes to place a psychiatrist in each divisional hospital, who should select the cases suitable for transfer to the neurological centers. The speaker felt that this was a mistake, as every psychiatrist does not possess the training and skill needed to diagnose nervous diseases and to enable him to select with certainty the cases that require special treatment. These psychoneuroses differ in no way from those that we are accustomed to meet in civil life, and these come into the hands of the neurologist for treatment rather than into the institutions to which the work of most psychiatrists is limited. The services of one psychiatrist in each center is, however, advisable, as a certain number of men with early mental disturbances are frequently admitted, and a psychiatrist can undertake the care of the exhaustion psychoses and certain purely emotional conditions which frequently develop after periods of severe fighting.

There should be also in each center arrangements for physical training, organized games, and modified drill by which the men who recover can be gradually hardened up for active service and their physical health and morale improved,

It was unnecessary, the speaker believed, to deal with the methods of treatment that have been adopted in the army centers. They are identical with those that have been found so successful by our French allies. They were referred to by previous speakers. In a word, the British rely upon suggestion and persuasion, and the careful sorting out of men who are unfit owing to physical and mental defects.

As to the nature of the cases which should be admitted to the Army neurological centres, the speaker said that the psychoneuroses may be roughly divided into hysteria, neurasthenia, — especially traumatic neurasthenia, — and such cases as fall into Dupré's emotional syndrome. We find, however, that a certain number of men who do not suffer from any of these conditions are frequently sent down for admission. Practically all combatant units contain men who are regarded by their officers as unfit for active soldiering or undesirable, and the easiest method of getting rid of them is on medical grounds. They should be, however, rigidly excluded from the neurological centers as they may have a bad moral effect on men suffering with genuine psychoneuroses and taint that healthy atmosphere which is one of the greatest assets in treating functional conditions.

Still more serious is the frequent error made in sending to the neurological centers men suffering from slight concussion only, due to being blown over by the bursting of a shell or being buried in a blown-in trench or dug-out. Many of these cases are certainly abnormally emotional and present symptoms we ordinarily see after concussion, but if they are given rest or light duty in the transport lines, or in the regimental aid post under the medical officer's direct supervision they are generally fit to return to duty within a few days. If, however, they are admitted among men with severe neurotic manifestations, it frequently happens that they develop by subconscious mimicry symptoms similar to those of the patients around them; for most men who have been subjected to a severe emotional strain become for a time abnormally suggestible and are consequently liable to develop any symptoms which are suggested to them by their surroundings. The speaker considered this one of the most important points to be emphasized, for his experience had been that when new and inexperienced officers are attached to divisions, the number of such unsuitable cases transferred to centers increases, and there is consequently a danger that the centers may do positive harm as well as render valuable service.

Professor Pierre Marie said that after hearing the very interesting reports which had been made, he had the impression that a certain number of our British and American colleagues had a very set idea on the subjet of commotion. They seemed to believe that unless there is a mark or injury on some part of the body, and especially the skull, the patient can be considered, if not a malingerer, at least a bad soldier, who is not loyally fulfilling his duty towards his country.

The French neurologists came to the same conclusion in the latter part of the first year of the war.

When, however, more cases had been seen the neurologists had to change their first ideas and no longer considered all cases of commotion as neuropaths or malingerers. The speaker was convinced that without any local lesions commotion might be caused by organic alterations of the nervous centers which can be observed by means now at hand, and especially, from a clinical point of view, lumbar puncture. Sometimes the cerebro-spinal liquid contains blood. In such cases it is evident that there is no gross lesion of the nervous centers. Sometimes it is simply xanthochromic, but the signification is the same. In the greater number of true commotion its aspect is normal, but shows a modified formula the clinical meaning of which is very important. This formula means that: —

1. The quantity of albumen in the cerebro-spinal liquid has increased; from 0.25 gram. to 1 gram. instead of from 0 15 to 0.25.

2. The number oi the cells is smaller, from 0.3 to 0.7., instead of from 1 to 2.

3. There is a slight tendency to hyperglucosis in a considerable number of cases. One must also take into consideration the more or less prolonged loss of consciousness which nearly always marks the beginning of the state of commotion. One will also frequently see labyrinthine troubles, and sometimes even gross lesions of the tympanum. All these objective signs can be explained only by admitting the existence of alterations of the nervous centers.

Mr. Lesene was right when he said that a commotion could be considered as a diffuse micro-traumatism, meaning that the traumatism involved only the microscopical elements. At the end of four or six weeks the formula of the cerebro-spinal liquid becomes practically normal again.

The cause of the state of commotion may be a change of pressure, but more probably the sudden shock, such as the displacement of air due to an explosion. The speaker in conclusion, expressed the conviction that although it is a good thing to punish malingerers and fakers, it would be a great injustice to overlook cases of organic commotion in which the patients are really wounded, even if their wounds are merely micro-traumatic.

